# Early Vitamin C and Thiamine Administration to Patients with Septic Shock in Emergency Departments: Propensity Score-Based Analysis of a Before-and-After Cohort Study

**DOI:** 10.3390/jcm8010102

**Published:** 2019-01-16

**Authors:** Tae Gun Shin, Youn-Jung Kim, Seung Mok Ryoo, Sung Yeon Hwang, Ik Joon Jo, Sung Phil Chung, Sung-Hyuk Choi, Gil Joon Suh, Won Young Kim

**Affiliations:** 1 Departm and nd t of Emergency Medicine, Samsung Medical Center, Sungkyunkwan University School of Medicine, Seoul 06351, Korea; taegunshin@skku.edu (T.G.S.); gerup@hanmail.net (S.Y.H.); drjij@skku.edu (I.J.J.); 2 Department of Emergency Medicine, Asan Medical Center, University of Ulsan College of Medicine, Seoul 05505, Korea; yjkim.em@gmail.com (Y.-J.K.); chrisryoo@naver.com (S.M.R.); 3 Department of Emergency Medicine, Yonsei University College of Medicine, Seoul 06273, Korea; emstar@naver.com; 4 Department of Emergency Medicine, Guro Hospital, Korea University Medical Center, Seoul 08308, Korea; kuedchoi@korea.ac.kr; 5 Department of Emergency Medicine, Seoul National University College of Medicine, Seoul 03080, Korea; suhgil@snu.ac.kr

**Keywords:** sepsis, septic shock, thiamine, vitamin C, resuscitation

## Abstract

Background: Intravenous vitamin C and thiamine administration may be a potential adjuvant therapy for septic shock. We aimed to investigate the impact of early vitamin C and thiamine administration in septic shock patients. Methods: This retrospective before-and-after cohort study used data extracted from the Korean Shock Society’s prospective septic shock registry. We compared 28-day and in-hospital mortality rates between patients treated with intravenous vitamin C (3 g/12 h or 1.5 g/6 h) and thiamine (200 mg/12 h) <6 h after shock recognition from July through December 2017 (*n* = 229) and control patients from October 2015 through June 2017 (*n* = 915) using propensity score matching. Results: The 28-day (18.3% vs. 17.5%; *p* = 0.76) and in-hospital (16.6% vs. 18.3%; *p* = 0.55) mortality rates did not differ between treatment and control groups, nor did 28-day (18.5% vs. 17.5%; *p* = 0.84) and in-hospital (16.7% vs. 18.4%; *p* = 0.54) mortality rates after matching. In the subgroup analysis, treatment was associated with lower in-hospital mortality rates in patients with albumin <3.0 mg/dL or a Sequential Organ Failure Assessment (SOFA) score >10. Conclusion: Early vitamin C and thiamine administration in patients with septic shock did not improve survival; however, administration could benefit conditions that are more severe, such as hypoalbuminemia or severe organ failure.

## 1. Introduction

Sepsis is a complex disease involving life-threatening organ dysfunction caused by a dysregulated host response to infection that remains associated with unacceptably high mortality rates [1]. Septic shock should be considered a medical emergency, and focus must be placed on timely intervention, including the early identification and treatment of infection through appropriate antimicrobial therapy and source control when applicable, as well as the reversal of hemodynamic instability through fluid resuscitation and vasopressor use, if necessary [2]. Despite these supportive therapies, morbidity and mortality rates remain high, suggesting the need for adjuvant therapies for inflammatory and oxidative stress in patients with septic shock. However, no agents to-date have been proven to definitely improve survival [3,4]. 

Vitamin C plays a role in mediating inflammation through antioxidant activity, and is an important co-factor/co-substrate for the synthesis of endogenous adrenaline, cortisol, and vasopressin [5]. Several clinical trials have reported the positive effects of vitamin C on sepsis or septic shock outcomes [6,7,8,9]. During sepsis, vitamin C prevents neutrophil-induced lipid oxidation and protects against endothelial barrier loss. Therefore, early intravenous supplementation for sepsis would be beneficial in preventing microcirculation loss and lipid oxidation [10]. Thiamine is also a key co-factor in glucose metabolism, adenosine triphosphate generation, and nicotinamide adenine dinucleotide phosphate production [7,11]. Considering acute consumption in the hypermetabolic state, thiamine supplementation might be a reasonable therapeutic adjunct for patients with sepsis, and has been added to reduce the risk of renal oxalate crystallization [12]. These findings led to a recent before-and-after study showing that sepsis treatment consisting of a combination of vitamin C, hydrocortisone, and thiamine prevented organ dysfunction and reduced mortality rates [6].

Although evidence is emerging that the parenteral administration of vitamin C may be a potential adjuvant therapy, the clinical effect on patients with septic shock and the optimal administration protocol remain to be determined. In this retrospective before-and-after study, we evaluated the efficacy of early vitamin C and thiamine administration for patients with septic shock who were treated with protocol-driven resuscitation bundle therapy in emergency departments (EDs).

## 2. Material and Methods

### 2.1. Study Design and Population

We performed a propensity score-based analysis of this before-and-after study in EDs at two teaching hospitals (hospital A: tertiary referral hospital in Seoul, Korea, with 75,000 annual ED visits; hospital B: tertiary referral hospital in Seoul, Korea, with 110,000 annual ED visits). Vitamin C and thiamine infusion was adopted as the routine adjunctive therapy for septic shock between July 2017 and December 2017. These two hospitals participated in a prospective multicenter septic shock registry of the Korean Shock Society, a collaborative research network that was constructed to improve sepsis management in 10 teaching hospitals throughout South Korea. The study was approved by the Institutional Review Board of Samsung Medical Center (IRB No.: 2018-08-003). The need for informed consent was waived given the study’s retrospective, observational, and anonymous nature.

In the septic shock registry of the Korean Shock Society, all adult patients (age ≥ 19 years) with septic shock who were diagnosed in an ED were enrolled prospectively since October 2015 [13,14,15]. Hospital A participated in this registry from the beginning, and Hospital B started enrolling patients in January 2016. Septic shock was defined as refractory hypotension requiring vasopressors despite adequate fluid therapy (20–30 mL/kg crystalloid solution), or hypoperfusion, which was defined as a blood lactate concentration ≥4 mmol/L in patients with suspected or confirmed infection [16,17,18]. Patients were excluded if they were in a “Do Not Attempt Resuscitation” state; if septic shock was recognized 6 h after arrival in the ED; if they were transferred from other hospitals and did not meet the inclusion criteria on ED arrival; or if they were transferred directly from the ED to other hospitals [15]. All participating hospitals of the Korean Shock Society treated patients with protocol-driven 6-hour septic shock bundle therapy [13,14,15].

In this study, the patients with septic shock enrolled from July to December 2017 were treated with vitamin C and thiamine and considered as the treatment group, whereas the patients enrolled from October 2015 to June 2017 were considered as the control group. Within 6 h of shock recognition, vitamin C and thiamine were mixed in 50- or 100-mL solution bags of 5% dextrose in water or normal saline and intravenously administered for 1 day (vitamin C, 3 g/12 h or 1.5 g/6 h; thiamine, 200 mg/12 h). Additional administrations of vitamin C and thiamine were performed according to the duty physician’s decision in the ED or intensive care unit (ICU). All patients with septic shock were treated with protocol-driven resuscitation bundle therapy based on the Surviving Sepsis Campaign guidelines, including initial crystalloid bolus infusions, blood culture, broad-spectrum antibiotics, vasopressors, lung-protective ventilation, glucocorticoids, and surgical intervention [2]. The study was approved by the Institutional Review Board of Samsung Medical Center (IRB No.: 2018-08-003). The need for informed consent was waived given the study’s retrospective, observational, and anonymous nature.

### 2.2. Data Collection

The case report form included standard definitions of 200 variables including clinical characteristics, therapeutic interventions, and outcomes of patients with septic shock [13,14,15]. All data were collected using standardized web-based report forms by research coordinators at each participating hospital and monitored by a quality management committee. To ensure data quality, the data were centrally reviewed at the coordinating hospital. From the registry, demographic characteristics, comorbidities, vital signs, suspected infection sources, blood culture findings, laboratory data, interventions (including antibiotics, initial fluid resuscitation, vasopressor use, mechanical ventilation, and renal replacement therapy), and outcomes were retrieved. Hypoalbuminemia was defined as an albumin level <3.0 g/dL. To calculate maximum Sequential Organ Failure Assessment (SOFA) and Acute Physiology and Chronic Health Evaluation (APACHE) II scores, the worst parameters within 24 h after ED arrival were used [19,20]. Organ dysfunction was defined as a SOFA score ≥2 points [1]. We also collected data on whether the included patients fulfilled the clinical criteria for septic shock according to the Third International Consensus definitions, defined as refractory hypotension requiring vasopressors to maintain a mean arterial pressure ≥65 mm Hg and a serum lactate level >2 mmol/L despite adequate volume resuscitation [1]. The primary outcome was 28-day mortality. Secondary outcomes were in-hospital mortality, hospital length of stay (LOS), intensive care unit (ICU) LOS, duration of mechanical ventilation, and need for renal replacement therapy. The datasets used and/or analyzed during the current study are available from the corresponding author by reasonable request.

### 2.3. Statistical Analyses

We present the data as median with interquartile range (IQR) or mean ± standard deviation for numerical data and numbers with percentages for categorical data. Continuous variables were compared using a Student’s *t*-test or the Wilcoxon rank-sum test, while categorical variables were compared using the chi-square test. We used propensity score matching to adjust for patient imbalances between the treatment and control groups using variables including age, sex, comorbidities, source of infection, laboratory test results (white blood cell count, hemoglobin level, platelet count, creatinine level, alanine aminotransferase level, albumin level, initial lactate level, and bacteremia), septic shock criteria meeting the Sepsis-3 consensus definition [1], adjunctive steroid use within 48 h, vasopressor use, use of mechanical ventilation, interventions for infection source control, maximum SOFA score in the initial 24 h, and APACHE II score. We performed 1-to-N matching with a caliper = 0.2 for each hospital cohort. All covariates were used for the matching variables. Balance was evaluated based on mean standardized differences and the generalized estimating equation approach. After propensity score matching, the primary and secondary outcomes were compared using generalized linear mixed models with the random effect of the hospital as a factor. For the overall unmatched cohort and subgroups, random-effects multivariate generalized linear mixed models were also used. Subgroups were defined according to age (>75 or ≤75 years), renal failure (initial creatinine >2.0 mg/dL or previous dialysis or creatinine ≤2.0 mg/dL without dialysis), malignancy (the presence of metastatic solid cancer or hematologic malignancy), albumin level (≥3.0 or <3.0 mg/dL), septic shock meeting the criteria of the Sepsis-3 consensus definition, SOFA score (>10 or ≤10 points), and adjunctive steroid use. Variables with a *p*-value < 0.2 were adjusted in the multivariate analysis. Mortality outcomes (28-day and in-hospital mortality rates) are described as odds ratio (OR) and 95% confidence interval (CI). All two-tailed *p*-values < 0.05 were considered statistically significant. The statistical analysis was executed using SAS version 9.4 (SAS Institute, Cary, NC, USA), R 3.4.3 (Vienna, Austria; http://www.R-project.org/), and STATA 15.0 (STATA Corporation, College Station, TX, USA) by independent biostatisticians. 

## 3. Results

### 3.1. Baseline Characteristics 

A total of 1144 patients with septic shock from the multicenter registry were analyzed (574 from hospital A, 570 from hospital B). Among them, 229 patients who received vitamin and thiamine infusions during their initial resuscitations were assigned to the treatment group (85 from hospital A, 144 from hospital B), whereas the 915 patients who did not receive infusions comprised the control group. The baseline characteristics and comparisons of the treatment and control groups are presented in Table 1. The median patient age was 67 years (IQR 58–75), and 713 patients (62.3%) were men. The most common infection focus was intra-abdominal infection (38.0%), followed by respiratory infection (23.4%). The median maximum SOFA score in 24 h was 8 (IQR, 5–11). The overall 28-day and in-hospital mortality rates were 17.6% and 17.9%, respectively. Between groups, there were statistically significant differences in suspected infection focus, presence of bacteremia, clinical criteria of septic shock according to the Sepsis-3 consensus definition, vasopressor use, and SOFA score. The treatment group showed more frequent bacteremia, clinical criteria of septic shock, and vasopressor use. The SOFA score was also higher in the treatment group. The baseline characteristics for each hospital are shown in Table S1. 

### 3.2. Outcomes

For the unmatched cohort, the 28-day mortality rate was 18.3% in the treatment group and 17.5% in the control group (*p* = 0.76; Table 2). The in-hospital mortality rates were 16.6% and 18.3%, respectively (*p* = 0.55). Other secondary outcomes also showed no significant differences. Propensity matching of all baseline variables for each hospital cohort revealed statistical balance between the two groups in hospitals A and B (Table S2). In the propensity-matched cohort, there were 227 and 527 patients in the treatment and control groups, respectively. There was no significant difference in 28-day mortality (18.5% vs. 17.5%, *p* = 0.84), in-hospital mortality (16.7% vs. 18.4%, *p* = 0.54), ICU LOS (4 (IQR, 3–8) vs. 4 (IQR, 3–7) days, *p* = 0.84), hospital LOS (14 (IQR, 9–22) vs. 13 (IQR, 8–23) days, *p* = 0.33), duration of mechanical ventilation (5.5 (IQR, 3.0–15.0) vs. 5.0 (IQR, 3.0–10.0) days, *p* = 0.63), or the need for renal replacement therapy (12.4% vs. 12.9%, *p* = 0.51).

After adjustment for confounding factors in the multivariate models, the adjusted OR was 0.86 (95% CI, 0.56–1.33; *p* = 0.51) for 28-day mortality and 0.69 (95% CI, 0.44–1.08; *p* = 0.11) for in-hospital mortality (Table 3). 

**Figure 1:**
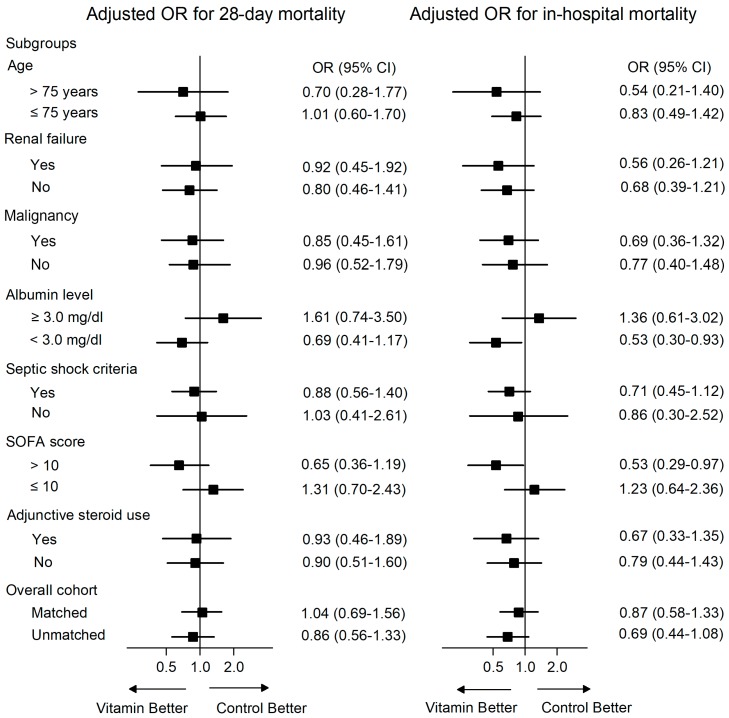
Random-effects multivariate analysis of 28-day and in-hospital mortality in unmatched subgroups.

In the subgroup analysis (Figure 1), treatment was significantly associated with lower in-hospital mortality rates among those with hypoalbuminemia (albumin < 3.0 mg/dL; adjusted OR, 0.53; 95% CI, 0.30–0.93; *p* = 0.02) and higher SOFA scores (>10 points; adjusted OR, 0.53; 95% CI, 0.29–0.97; *p* = 0.03). In patients with albumin <3.0 mg/dL, the 28-day mortalities were 21.9% vs. 26.2% (treatment vs. control, *p* = 0.24) and in-hospital mortalities were 19.2% vs. 26.9% (*p* = 0.18) (Table 4). In patients with SOFA score >10 points, the 28-day mortalities were 38.1% vs. 30.1% (*p* = 0.24) and in-hospital mortalities were 42.2% vs. 28.8% (*p* = 0.04). 

On the other hand, there was no significant association with mortality in patients with albumin ≥3.0 mg/dL or SOFA scores ≤10. For other subgroups according to renal failure, malignancy, the criteria of the Sepsis-3 consensus definition, and adjunctive steroid use, no significant beneficial or harmful effects on mortality were observed.

## 4. Discussion

Despite the theoretical benefits of vitamin C and thiamine administration, in this propensity score-based analysis of a before-and-after cohort study, combination therapy during the initial resuscitation of patients with septic shock did not significantly impact mortality. Early vitamin C and thiamine therapy had a beneficial effect on survival only in a subgroup of patients with hypoalbuminemia or severe organ failure. 

Several preliminary studies have reported on the adjunctive use of vitamin C or thiamine for patients with sepsis or septic shock. Fowler et al. performed a pilot study of 24 patients with severe sepsis and septic shock randomized into placebo (*n* = 8), low-dose intravenous vitamin C (50 mg/kg; *n* = 8), and high-dose vitamin C (200 mg/kg; *n* = 8) groups [9]. Vitamin C significantly reduced the levels of inflammatory biomarkers, including C-reactive protein and procalcitonin. Zabet et al. conducted a randomized trial of intravenous vitamin C (100 mg/kg/day) in patients with septic shock in the ICU (*n* = 28) [8]. In this study, the mean dose and duration of norepinephrine administration were significantly lower than those in the treatment group. However, in our study analyzing data from a relatively larger population from two EDs, the early vitamin C and thiamine therapy was not associated with mortality rate improvements. The possible reasons for this discrepancy are, first, that the vitamin C administration duration was relatively short. A recent study on vitamin C pharmacokinetics showed that sustained vitamin C supplementation for more than two or three days is needed to prevent hypovitaminosis in critically ill patients, despite high-dose infusions [21]. Second, the overall mortality rate was relatively lower than that in ICU studies of septic shock showing control group mortality rates >40% [8,22]. Third, a single-center before-and-after study by Marik et al. evaluating the combined use of vitamin C, thiamine, and hydrocortisone showed a marked reduction in mortality rates and follow-up SOFA scores compared with those of matched controls [6]. The researchers also suggested synergistic effects of combining vitamin C and steroids in this and an experimental study, which require further validation [22]. Finally, patients in the treatment group had organ failure and septic shock that was more severe, although this was adjusted using the statistical methods. 

We observed significant associations between treatment and in-hospital mortality in subgroups with severe organ failure or hypoalbuminemia. Vitamin C depletion might be associated with multiple organ failure [23], and patients with higher SOFA scores could have lower vitamin concentrations requiring supplementation. Hypoalbuminemia can be affected by sepsis severity, organ failure, and chronic malnutrition, and might also be a risk factor for vitamin depletion [13]. Therefore, our results suggest a beneficial effect of vitamin combination therapy in some candidates. Further studies are required to identify the risk factors associated with vitamin deficiency in patients with septic shock as well as subgroups of patients who may benefit from vitamin supplementation. Larger trials are needed to confirm these results and evaluate the ideal dosage and duration strategies. 

This study has some limitations. First, this was a retrospective observational study and the primary objective of the original prospective registry did not involve the use of vitamins. The study was conducted in the EDs of two tertiary academic institutions; thus, the ability to generalize these results in other settings is limited. Second, the vitamin treatment was not randomized, and some patients did not receive the combination therapy after adoption of the adjunctive use of vitamins. There might be some bias, although we compared outcomes with the maximal adjustment of potential observed variables. Third, vitamin C and thiamine combination therapy was limited in the early resuscitation period, and the treatment duration might have been too short to prove any effect on outcomes. Fourth, we could not compare other outcomes, including the incidence of acute kidney injury, duration of vasopressor infusion, or incidence of delirium, because of limited data.

## 5. Conclusions

Intravenous vitamin C and thiamine infusion during the initial resuscitation period in patients with septic shock was not associated with improved survival. Considering the individual variability of patients with sepsis, its use could be beneficial in a subgroup of patients, such as those with hypoalbuminemia or severe organ failure. However, further prospective studies are needed to clarify the clinical implications of our findings.

## Figures and Tables

**Table 1:** Baseline characteristics of the unmatched cohort.

Variable	Overall (*n* = 1144)	Treatment Group (*n* = 229)	Control Group (*n* = 915)	*p*
Age (years)	67 (58–75)	67 (58–76)	67 (60–75)	0.91
Sex, male	713 (62.3)	136 (59.4)	577 (63.1)	0.30
Comorbidities				
Hypertension	412 (36.1)	89 (38.9)	323 (35.3)	0.31
Diabetes	335 (29.3)	71 (31.0)	264 (28.9)	0.52
Cardiac disease	152 (13.3)	24 (10.5)	128 (13.4)	0.16
Chronic lung disease	94 (8.2)	17 (7.4)	77 (8.4)	0.62
Chronic renal disease	78 (6.8)	15 (6.6)	63 (6.9)	0.85
Chronic liver disease	180 (15.7)	35 (15.3)	145 (15.9)	0.83
Hematologic malignancy	113 (9.9)	19 (8.3)	94 (10.3)	0.37
Metastatic solid cancer	364 (31.8)	72 (31.4)	292 (31.9)	0.89
Suspected infection focus				0.04
Respiratory infection	268 (22.4)	41 (17.9)	227 (24.8)	
Urinary tract infection	158 (13.8)	41 (17.9)	117 (12.8)	
Intra-abdominal infection	435 (38.0)	85 (37.1)	350 (38.3)	
Others or unknown	283 (24.7)	62 (27.1)	221 (24.2)	
Laboratory tests				
White blood cell count, ×10^3^/μL	9.6 (4.5–16.3)	8.1 (4.1–15.1)	9.9 (4.6–16.9)	0.17
Hemoglobin (g/dL)	10.7 (9.0–12.4)	10.6 (9.0–12.2)	10.8 (9.0–12.5)	0.33
Platelet count (×10^3^/μL)	137 (66–215)	132 (72–183)	138 (65–225)	0.30
Creatinine (mg/dL)	1.3 (0.9–2.0)	1.4 (1.0–2.2)	1.3 (0.9–2.0)	0.06
ALT (U/L)	28 (16–57)	30 (16–57)	28 (16–57)	0.85
Albumin (g/dL)	2.9 (2.4–3.4)	2.8 (2.4–3.3)	3.0 (2.5–3.4)	0.01
Initial lactate (mmol/L)	3.6 (2.0–5.5)	3.4 (2.1–5.7)	3.7 (1.9–5.5)	0.72
Blood culture—positive	484 (42.3)	367 (40.1)	117 (51.1)	<0.01
Septic shock criteria, Sepsis-3 consensus definition	593 (51.8)	152 (66.4)	441 (48.2)	<0.01
Adjunctive steroid use within 48 h	287 (25.1)	62 (27.1)	225 (24.6)	0.43
Vasopressor use	966 (84.4)	217 (94.8)	749 (81.9)	<0.01
Mechanical ventilation	328 (17.9)	67 (39.3)	261 (28.5)	0.82
Interventions for source control	332 (29.0)	58 (25.3)	274 (30.0)	0.16
Maximum SOFA score in 24 h	8 (5–11)	9 (6–12)	8 (5–11)	<0.01
APACHE II score	20 (15–27)	27 (21–52)	27 (20–56)	0.99

Data are shown as median (interquartile range) or as *n* (%). ALT: alanine aminotransferase; SOFA: Sequential Organ Failure Assessment; APACHE: Acute Physiology and Chronic Health Evaluation.

**Table 2:** Comparisons of primary and secondary outcomes.

	Overall Cohort			Propensity-Matched Cohort		
	Treatment (*n* = 229)	Control (*n* = 915)	*p*	Treatment (*n* = 227)	Control (*n* = 527)	*p*
28-day mortality	42 (18.3)	160 (17.5)	0.76	42 (18.5)	92 (17.5)	0.84
In-hospital mortality	38 (16.6)	167 (18.3)	0.55	38 (16.7)	97 (18.4)	0.54
ICU LOS (days)	4 (3–8)	4 (3–8)	0.70	4 (3–8)	4 (3–7)	0.84
Hospital LOS (days)	14 (9–22)	13 (8–23)	0.49	14 (9–22)	13 (8–23)	0.33
Duration of mechanical ventilation	6.0 (3.0–15.0)	6.0 (3.0–12.0)	0.61	5.5 (3.0–15.0)	5.0 (3.0–10.0)	0.63
New use of renal replacement therapy	28 (12.3)	106 (11.9)	0.87	28 (12.4)	66 (12.9)	0.51

Data are shown as median (interquartile range) or as *n* (%). ICU: intensive care unit; LOS: length of stay.

**Table 3:** Random-effects multivariate analysis of 28-day and in-hospital mortality.

Variable	28-Day Mortality			In-Hospital Mortality		
	Adjusted OR	95% CI	*p*	Adjusted OR	95% CI	*p*
Vitamin treatment	0.86	0.56–1.33	0.51	0.69	0.44–1.08	0.11
Infection focus						
Respiratory	Reference			Reference		
UTI	0.52	0.26–1.04	0.06	0.36	0.18–0.74	0.01
Abdomen	0.73	0.45–1.20	0.22	0.52	0.32–0.86	0.01
Other	1.08	0.67–1.74	0.75	0.77	0.47–1.25	0.29
WBC count (>12,000/μL)	1.25	0.87–1.79	0.22	1.33	0.93–1.92	0.12
Creatinine (>2.0 mg/dL)	0.93	0.62–1.40	0.73	0.84	0.55–1.29	0.43
Albumin (<3.0 mg/dL)	3.00	2.05–4.39	<0.01	3.29	2.13–5.06	<0.01
Blood culture positive	0.51	0.34–0.76	<0.01	0.67	0.45–1.01	0.06
Septic shock criteria	2.16	1.36–3.43	<0.01	2.88	1.75–4.72	<0.01
Vasopressor use	0.25	0.14–0.47	<0.01	0.22	0.11–0.42	<0.01
Source control interventions	0.58	0.36–0.93	0.02	0.55	0.34–0.88	0.01
Maximum 24-h SOFA score	1.30	1.23–1.37	<0.01	1.31	1.23–1.38	<0.01

CI: confidence interval; OR: odds ratio; SOFA: Sequential Organ Failure Assessment; UTI: urinary tract infection; WBC: white blood cell.

**Table 4:** Crude 28-day and in-hospital mortality.

Subgroups	28-Day Mortality		In-Hospital Mortality	
	Treatment	Control	Treatment	Control
Age (years)				
>75	20.7 (12/58)	17.5 (42/240)	20.0 (11/58)	17.9 (43/240)
≤75	17.5 (30/171)	17.5 (118/675)	15.8 (27/171)	18.4 (124/675)
Renal failure				
Yes	29.7 (19/64)	25.8 (59/229)	25.0 (16/64)	26.2 (60/229)
No	13.9 (23/165)	14.7 (101/686)	13.3 (22/165)	15.6 (107/686)
Malignancy				
Yes	24.1 (22/91)	23.7 (91/384)	22.0 (20/91)	24.0 (92/384)
No	14.5 (20/138)	13.0 (69/531)	13.0 (13/138)	14.1 (75/531)
Albumin (mg/dL)				
≥3.0	13.6 (12/88)	9.0 (42/465)	12.5 (11/88)	9.9 (46/465)
<3.0	21.3 (30/141)	26.2 (118/450)	19.2 (27/141)	26.9 (121/450)
Septic shock criteria				
Yes	23.0 (35/152)	24.7 (109/441)	21.7 (33/152)	27.2 (120/441)
No	9.1 (7/77)	10.8 (51/474)	6.5 (5/77)	9.9 (47/474)
SOFA score				
>10	30.1 (22/73)	38.7 (89/230)	28.8 (21/73)	42.2 (97/230)
≤10	12.8 (20/156)	10.4 (71/685)	10.9 (17/156)	10.2 (70/685)
Adjunctive steroid use				
Yes	32.3 (20/62)	30.7 (69/225)	29.0 (18/62)	33.3 (75/225)
No	13.2 (22/167)	13.2 (91/690)	12.0 (20/167)	13.3 (92/690)

Data are shown as % (*n*). SOFA: Sequential Organ Failure Assessment.

## Supplementary Materials

The following are available online at http://www.mdpi.com/2077-0383/8/1/102/s1, Table S1: Baseline Characteristics of the Unmatched Cohort by Hospital, Table S2: Baseline Characteristics of the Propensity-Matched Cohort by Hospital.
