# Supplementary material for: Early Vitamin C and Thiamine Administration to Patients with Septic Shock in Emergency Departments: Propensity Score-Based Analysis of a Before-and-After Cohort Study
Source: J Clin Med. 2019 Jan 16;8(1):102. doi: 10.3390/jcm8010102 (PMC6352246; doi:10.3390/jcm8010102)
Supplement: Supplementary file 1 [file jcm-08-00102-s001.pdf]

Table S1. Baseline Characteristics of the Unmatched Cohort by Hospital.

Variable	Hospital A			Hospital B		
	Treatment Group (*n* = 85)	Control Group (*n* = 489)	*P*	Treatment Group (*n* = 144)	Control Group (*n* = 426)	*P*
Age, years	69 (60–76)	67 (58–75)	0.18	65 (58–74)	67 (59–76)	0.27
Sex, male	51 (60.0)	305 (62.4)	0.67	85 (59.0)	272 (63.9)	0.30
Comorbidities						
Hypertension	37 (43.5)	187 (38.2)	0.35	52(36.1)	136 (31.9)	0.35
Diabetes	29 (34.1)	159 (32.5)	0.77	42 (29.2)	105 (24.7)	0.28
Cardiac disease	11 (12.9)	79 (16.2)	0.45	13 (9.0)	49 (11.5)	0.40
Chronic lung disease	5 (5.9)	36 (7.4)	0.62	12 (8.3)	41 (9.6)	0.64
Chronic renal disease	6 (7.1)	36 (7.4)	0.92	9 (6.3)	27 (6.3)	0.97
Chronic liver disease	13 (15.3)	77 (15.8)	0.91	22 (15.3)	68 (16.0)	0.84
Hematologic malignancy	8 (9.4)	62 (12.7)	0.39	11 (7.6)	32 (7.5)	0.96
Metastatic solid cancer	21 (24.7)	134 (27.4)	0.60	51 (35.4)	158 (37.1)	0.71
Suspected infection focus			0.20			0.11
Respiratory infection	13 (15.3)	123 (25.2)		28 (19.4)	104 (24.4)	
Urinary tract infection	17 (20.0)	72 (14.7)		24 (16.7)	45 (10.6)	
Intra-abdominal infection	33 (38.8)	174 (35.6)		52 (36.1)	176 (41.3)	
Others or unknown	22 (25.9)	120 (24.5)		40 (27.8)	101 (23.7)	
Laboratory tests						
WBC count (×10³/μL)	9.4 (2.8–15.1)	9.3 (3.6–16.3)	0.64	7.8 (4.6–14.9)	10.4 (5.3–17.2)	0.06
Hemoglobin (g/dL)	10.3 (8.8–12.0)	10.9 (9.0–12.5)	0.16	10.6 (9.0–12.3)	10.7 (9.0–12.4)	0.99
Platelet count (×10³/μL)	113 (65–167)	131 (60–226)	0.25	142 (76–191)	146 (69–222)	0.48
Creatinine (mg/dL)	1.4 (0.9–2.5)	1.1 (0.8–1.8)	<0.01	1.2 (0.9–2.0)	1.3 (0.9–2.1)	0.82
ALT (U/L)	31 (15–57)	28 (17–54)	0.90	29 (16–57)	28 (15–59)	0.84
Albumin (g/dL)	3.0 (2.7–3.5)	3.3 (2.9–3.7)	0.01	2.6 (2.3–3.0)	2.6 (2.1–3.0)	0.15
Initial lactate (mmol/L)	4.1 (2.7–6.3)	4.1 (2.3–5.6)	0.37	3.0 (2.0–5.4)	3.1 (1.7–5.4)	0.51
Blood culture positive	54 (63.5)	186 (38.0)	<0.01	63 (43.8)	181 (42.5)	0.79
Septic shock criteria, Sepsis-3 consensus definition	65 (76.5)	243 (49.7)	<0.01	87 (60.4)	198 (46.5)	<0.01
Adjunctive steroid use	35 (41.2)	133 (27.2)	<0.01	27 (18.8)	92 (21.6)	0.46
Vasopressor use	81 (95.3)	367 (75.1)	<0.01	136 (94.4)	382 (89.7)	0.08
Mechanical ventilation	32 (37.7)	140 (28.6)	0.09	35 (24.3)	121 (28.4)	0.34
Source control interventions	25 (29.4)	158 (32.3)	0.59	33 (22.9)	116 (27.2)	0.30
Maximum 24-h SOFA score	10 (8–13)	8 (5–11)	<0.01	8 (6–10)	7 (5–11)	0.29
APACHE II score	27 (23–33)	23 (18–29)	<0.01	17 (11–22)	17 (13–22)	0.29

Data are shown as median (interquartile range) or *n* (%). ALT, alanine aminotransferase; APACHE, Acute Physiology and Chronic Health Evaluation; SOFA, Sepsis-related Organ Failure Assessment; WBC, white blood cell.

Table S2. Baseline Characteristics of the Propensity-Matched Cohort by Hospital.

Variable	Hospital A			Hospital B		
	Treatment	Control	P	Treatment	Control	P

	Group (*n* = 85)	Group (*n* = 256)		Group (*n* = 142)	Group (*n* = 271)	
Age, years	69 (60–76)	68 (58–76)	0.69	65 (58–75)	66 (57–75)	0.94
Sex, male	51 (60.0)	151 (59.0)	0.80	84 (59.2)	161 (59.4)	0.83
Comorbidities						
Hypertension	37 (43.5)	107 (41.8)	0.97	51 (35.9)	94 (34.7)	0.94
Diabetes	29 (34.1)	83 (32.4)	0.64	40 (28.2)	71 (26.2)	0.66
Cardiac disease	11 (12.9)	32 (12.5)	0.63	13 (9.2)	25 (9.2)	0.90
Chronic lung disease	5 (5.9)	15 (5.9)	0.75	11 (7.8)	23 (8.5)	0.80
Chronic renal disease	6 (7.1)	21 (8.2)	0.97	9 (6.3)	16 (5.9)	0.76
Chronic liver disease	13 (15.3)	38 (14.8)	0.87	21 (14.8)	43 (15.9)	0.85
Hematologic malignancy	8 (9.4)	21 (8.2)	0.60	11 (7.8)	20 (7.4)	0.89
Metastatic solid cancer	21 (24.7)	71 (27.7)	0.83	50 (35.2)	90 (33.2)	0.78
Suspected infection focus			0.99			0.86
Respiratory infection	13 (15.3)	43 (16.8)		27 (19.0)	53 (19.6)	
Urinary tract infection	17 (20.0)	48 (18.8)		23 (16.2)	40 (14.8)	
Intra-abdominal infection	33 (38.8)	99 (38.7)		52 (36.6)	103 (38.0)	
Others or unknown	22 (25.9)	66 (25.8)		40 (28.2)	75 (27.7)	
Laboratory tests						
WBC count (×10³/μL)	9.4 (2.8–15.1)	8.5 (2.4–15.7)	0.73	7.8 (4.6–15.0)	10.4 (4.9–16.7)	0.87
Hemoglobin (g/dL)	10.3 (8.8–12.0)	10.6 (8.7–12.2)	0.86	10.6 (9.0–12.4)	10.7 (9.0–12.7)	0.74
Platelet count (×10³/μL)	113 (65–167)	122 (46–225)	0.56	142 (77–189)	142 (66–204)	0.87
Creatinine (mg/dL)	1.4 (0.9–2.5)	1.3 (1.0–2.1)	0.93	1.2 (0.9–2.0)	1.4 (0.9–2.3)	0.75
ALT (U/L)	31 (15–57)	28 (18–55)	0.84	30 (16–58)	28 (16–59)	0.90
Albumin (g/dL)	3.0 (2.7–3.5)	3.2 (2.7–3.5)	0.75	2.6 (2.3–3.0)	2.6 (2.1–3.1)	0.78
Initial lactate (mmol/L)	4.1 (2.7–6.3)	3.5 (2.2–5.5)	0.90	3.0 (2.0–5.4)	3.1 (1.8–5.3)	0.93
Blood culture positive	54 (63.5)	134 (52.3)	0.65	62 (43.7)	119 (43.9)	0.83
Septic shock criteria, Sepsis-3 consensus definition	65 (76.5)	176 (68.8)	0.79	85 (59.9)	155 (57.2)	0.81
Adjunctive steroid use within 48 h	35 (41.2)	80 (31.3)	0.77	27 (19.0)	53 (19.6)	0.92
Vasopressor use	81 (95.3)	240 (93.8)	1.00	134 (94.4)	256 (94.5)	0.86
Mechanical ventilation	32 (37.7)	79 (30.9)	0.76	34 (23.9)	65 (24.0)	0.87
Source control interventions	25 (29.4)	85 (33.2)	0.66	33 (23.2)	63 (23.3)	0.87
Maximum 24-h SOFA score	10 (8–13)	9 (7–12)	0.68	8 (6–10)	7 (5–11)	0.69
APACHE II score	27 (23–33)	25 (20 -32)	0.54	17 (11–22)	17 (12–22)	0.82

Data are shown as median (interquartile range) or *n* (%). ALT, alanine aminotransferase; APACHE, Acute Physiology and Chronic Health Evaluation; SOFA, Sepsis-related Organ Failure Assessment; WBC, white blood cell.
